# Flow Diversion for the Treatment of MCA Bifurcation Aneurysms—A Single Centre Experience

**DOI:** 10.3389/fneur.2017.00020

**Published:** 2017-02-02

**Authors:** Pervinder Bhogal, Muhammad AlMatter, Hansjörg Bäzner, Oliver Ganslandt, Hans Henkes, Marta Aguilar Pérez

**Affiliations:** ^1^Neuroradiologic Clinic, Klinikum Stuttgart, Stuttgart, Germany; ^2^Neurologic Clinic, Klinikum Stuttgart, Stuttgart, Germany; ^3^Neurosurgical Clinic, Klinikum Stuttgart, Stuttgart, Germany; ^4^Medizinische Fakultät der Universität Duisburg-Essen, Essen, Germany

**Keywords:** flow diverter, pipeline embolization device, MCA aneurysm, MCA bifurcation, Stents, P64

## Abstract

**Background:**

Intracranial aneurysms located at the bifurcation of the middle cerebral artery (MCA) can often be challenging for the neurointerventionalist. We aimed to evaluate the efficacy and safety of flow diverting stents (FDS) in the treatment of these aneurysms.

**Materials and methods:**

We retrospectively reviewed our prospectively maintained database to collect information for all patients with unruptured saccular bifurcation MCA aneurysms treated with FDS between January 2010 and January 2016. In addition to demographic data, we recorded the location, aneurysm characteristics, previous treatments, number and type of FDS, complications, and clinical and angiographic follow-up.

**Results:**

Our search identified 13 patients (7 males) with an average age of 61.7 years (47–74 years). All patients had a single bifurcation aneurysm of the MCA, and none of the aneurysms were acutely ruptured. The average fundus size of the saccular aneurysms was 3 mm (range 1.5–10 mm). Follow-up studies were available for 12 patients. Based on the most recent follow-up angiograms, six aneurysms (50%) were totally occluded; five aneurysms (41.7%) showed only a small remnant; and one aneurysm (8.3%) remained unchanged. One patient suffered from an ischemic stroke with resultant permanent hemiparesis (mRS 3). In another case, there was an in-stent thrombosis during the intervention, which resolved upon intra-arterial infusion of Eptifibatide (mRS 0). There were no intra-operative vessel or aneurysm ruptures and no mortalities. Angiography of the covered MCA branches showed no change in the caliber or flow of the vessel in six (50%), a reduction in caliber in five (41.7%), and a complete occlusion in one (8.3%). All caliber changes and occlusions of the vessels were asymptomatic.

**Conclusion:**

In our series, 91.7% of treated MCA bifurcation aneurysms were either completely occluded or showed only a small remnant with a good safety profile. Flow diversion of MCA bifurcation aneurysms should be considered as an alternative treatment strategy when microsurgical clipping or alternative endovascular treatment options are not feasible.

## Introduction

Since the publication of the International Subarachnoid Aneurysm Trial, intracranial aneurysms are being increasingly treated *via* the endovascular approach as an alternative for craniotomy and surgical clipping (1). Aneurysms of the middle cerebral artery (MCA) remain, however, challenging for the neurointerventionist as they most commonly arise at a bifurcation of the vessel and frequently have a wide neck that can incorporate one or more branches (2), rendering traditional coiling difficult and necessitating the use of adjunctive devices such as balloon remodeling (3–7) or stent-assisted coiling (8–12). Over the last few years, flow diversion has proved to be a feasible and efficacious approach for the treatment of sidewall and dissecting aneurysms (13–19) even though long-term clinical data are still unavailable. The role of flow diverter stents (FDS) in treating bifurcation aneurysms remains, however, unclear. In this report, we review our experience in treating MCA bifurcation aneurysms with flow diversion as the primary approach or secondary therapy after previous coiling or clipping with subsequent recanalization/aneurysm residual.

## Patients and Methods

### Population

We retrospectively reviewed our prospectively maintained database to identify patients with aneurysms of the MCA bifurcation who were treated with flow diversion. Records were made of demographic data, clinical presentation, location and morphology of the aneurysms, the endovascular procedure, the postoperative complications, and the latest angiographic and clinical follow-up.

### Definition of MCA Bifurcation Aneurysm

We chose to categorize aneurysms arising at the first main division of the M1 trunk and those arising at an early division of a dominant superior or inferior trunk as MCA bifurcation aneurysms. Aneurysms of the M1 segment and distal to the MCA bifurcation were excluded.

### Endovascular Treatment

Informed consent was obtained prior to the intervention in all patients. A loading dose of two antiplatelet agents (aspirin 100 mg per day and clopidogrel 75 mg per day) was administered in every case, and the adequacy of the antiplatelet therapy was measured using the Multiplate Analyzer (Roche, Germany). Patients found resistant to clopidogrel received 2 × 90 mg ticagrelor daily. All therapeutic interventions were performed under general anesthesia. Arterial access was carried out through a standard 6Fr right common femoral route in all cases. A bolus dose of heparin (5,000 IU) was administered after securing the introducer sheath followed by repeat bolus doses every hour to maintain the activated clotting time between 2 and 2.5 times the baseline. The post-procedural antiplatelet regimen consisted of clopidogrel/ticagrelor continued for 12 months following treatment and aspirin continued for life.

A pipeline embolization device (PED) was used in one case (Covidien, Irvine, CA, USA), the p64 flow modulation device (Phenox, Bochum, Germany) was used in all other cases. The choice of flow diverter was down to the operator, and we only have the p64 and PED available in our department. The PED is one of the most widely studied devices and is made of 25% platinum and 75% nickel–cobalt–chromium alloy with a porosity of 65–70%. It is available in a variety of different sizes and diameters and multiple telescoped PED’s can be used to alter the porosity (20). The p64 is a braided flow diverting stent composed of 64 nitinol wires. Two platinum wires are wrapped around the shaft and assist in the radio opacity of the device. The 64 wires are grouped into 8 bundles proximally, with each bundle consisting of 8 wires. A radio-opaque marker is attached to end of each of these bundles. The porosity of the device is 51–60%. The p64 is unique amongst flow diverters in that it is mechanically detached and can be resheathed even after complete deployment.

### Clinical and Angiographic Follow-up

All patients were evaluated clinically and neurologically prior to the treatment and during the postoperative hospital stay, another clinical and neurological evaluation was performed at every follow-up as well. Initial follow-up catheter angiography was performed at 3 months. The extent of the aneurysmal occlusion was graded as:

- 1—total occlusion, no contrast filling of the aneurysm sac (Raymond Roy I)
- 2—subtotal occlusion, minor residual sac filling or neck remnant (Raymond Roy II)
- 3—incomplete occlusion, substantial residual sac filling (Raymond Roy III)
- 4—unchanged, patent aneurysmal sac with constant morphology compared to the pretreatment angiogram.

## Results

### Population

There were 13 patients matching our inclusion criteria (7 males) with an average age of 60 years (42–76 years). The treated aneurysms were equally distributed between right and left. Three patients had a second aneurysm at a different location, one patient had two and another patient had four other intracranial aneurysms located elsewhere. All aneurysms were saccular with a fundus size ranging from 1.5 to 10 mm (average fundus size 3 mm) and with the exception of one case all aneurysms had a dome/neck ratio of less than 1.5. Twelve aneurysms were located at the fist main MCA bifurcation (early bifurcation in two cases), one aneurysm was located at the bifurcation of each of the superior and inferior trunk. All patients were treated on an elective basis. The two fusiform aneurysms were previously clipped with incomplete occlusion. Four of the saccular aneurysms were previously treated by other modality (three were coiled and in one case there was an enlarging neck remnant after clipping and then coiling). The results are summarized in Table 1.

**Table 1:** **Demographics, aneurysm characteristics, clinical, and radiological follow-up**.

Patient no.	Gender	Age	Side	Dome (mm)	Neck (mm)	Previous treatments	FDS (no. × type)	Occlusion	Covered branch	Complications	Change in baseline mRS
1	m	60	L	2	2	Surgery, coils	1 × p64	2, RRC II	Unchanged	N	N
2	m	64	R	2	2	N	1 × p64	1, RRC I	Asymptomatic occlusion	N	N
3	f	47	L	1.5	1.5	Coils	1 × p64	1, RRC I	Unchanged	N	N
4	f	50	L	2	2	Coils	1 × p64	1, RRC I	Reduction in caliber	N	N
5	f	60	R	4	4	N	1 × p64	2, RRC II	Reduction in caliber	N	N
6	m	58	R	2	3	N	1 × p64	2, RRC II	Reduction in caliber	N	N
7	m	60	L	3	4	N	1 × p64	1, RRC I	Unchanged	N	N
8	m	76	R	10	3.5	N	1 × p64	2, RRC II	Reduction in caliber	N	N
9	f	74	R	4	1.5	N	1 × p64	4	Reduction in caliber	N	N
10	m	58	L	2	2	N	1 × p64	NA	NA	N	N
11	m	71	L	2	3	N	1 × p64	2, RRC II	Unchanged	Stroke	Y (mRS 3)
12	f	59	L	3	1	N	1 × p64	1, RRC I	Unchanged	Asymptomatic thrombosis	N
13	f	65	R	2	2	N	1 × pipeline embolization device	1, RRC I	Unchanged	N	N

### Feasibility

The delivery of the FDS was unproblematic in all cases. A single flow diverter was used in all cases. A PED was used in one case; all other patients were treated using the p64 flow modulation device.

### Angiographic Follow-up

**Figure 1:**
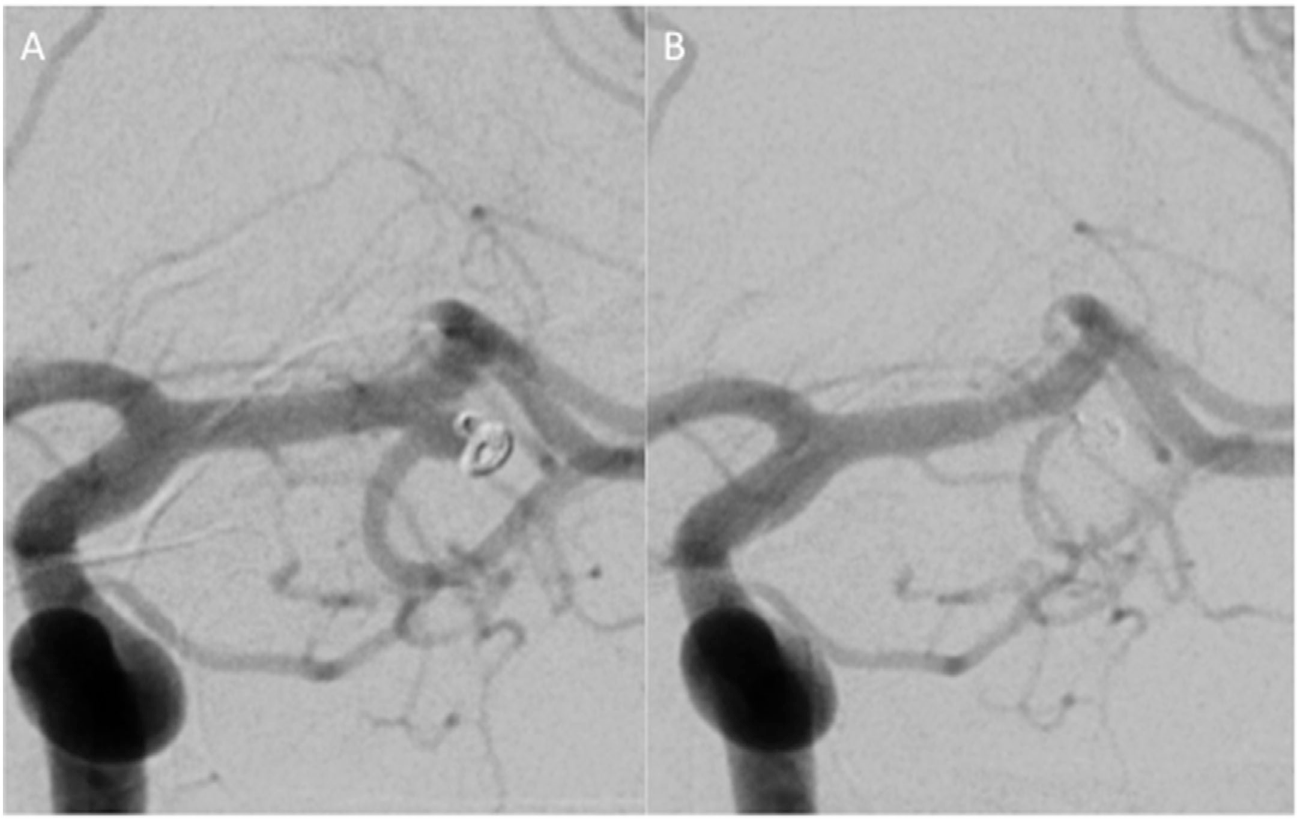
**A small neck recurrence after a previous coiling (A)**. Repeat treatment using coils would have necessitated a stent into the inferior branch, and therefore, it was felt an alternative strategy would be to place a single p64 FDS into the superior middle cerebral artery trunk. A follow-up angiogram performed 3 months later **(B)** showed on contrast enhancement of the aneurysm and a reduction in the caliber of the size of the inferior trunk but with persistent anterograde flow. The patient was neurologically intact, and there were no clinical consequences of the vessel modification.

Follow-up studies were available for 12 patients with first follow-up catheter angiography performed at mean 3.1 months after treatment. Delayed angiography was performed at mean 15.8 months. There was complete aneurysmal occlusion in six cases (50%) (Figure 1). Near-complete occlusion with only a neck remnant was achieved in a further five cases (41.7%). A single case remained unchanged in our series; therefore, complete or near-complete occlusion was achieved in 91.7% of our cases.

The covered branches remained unchanged in caliber and flow in six cases (50%). In five cases, there was a reduction in the caliber of the covered branch but the vessel remained patent with anterograde flow (Figure 1). There was a single case of asymptomatic occlusion of the covered branch (8.3%). All the cases where the covered vessel caliber was reduced or completely occluded were clinically asymptomatic.

### Complications

**Figure 2:**
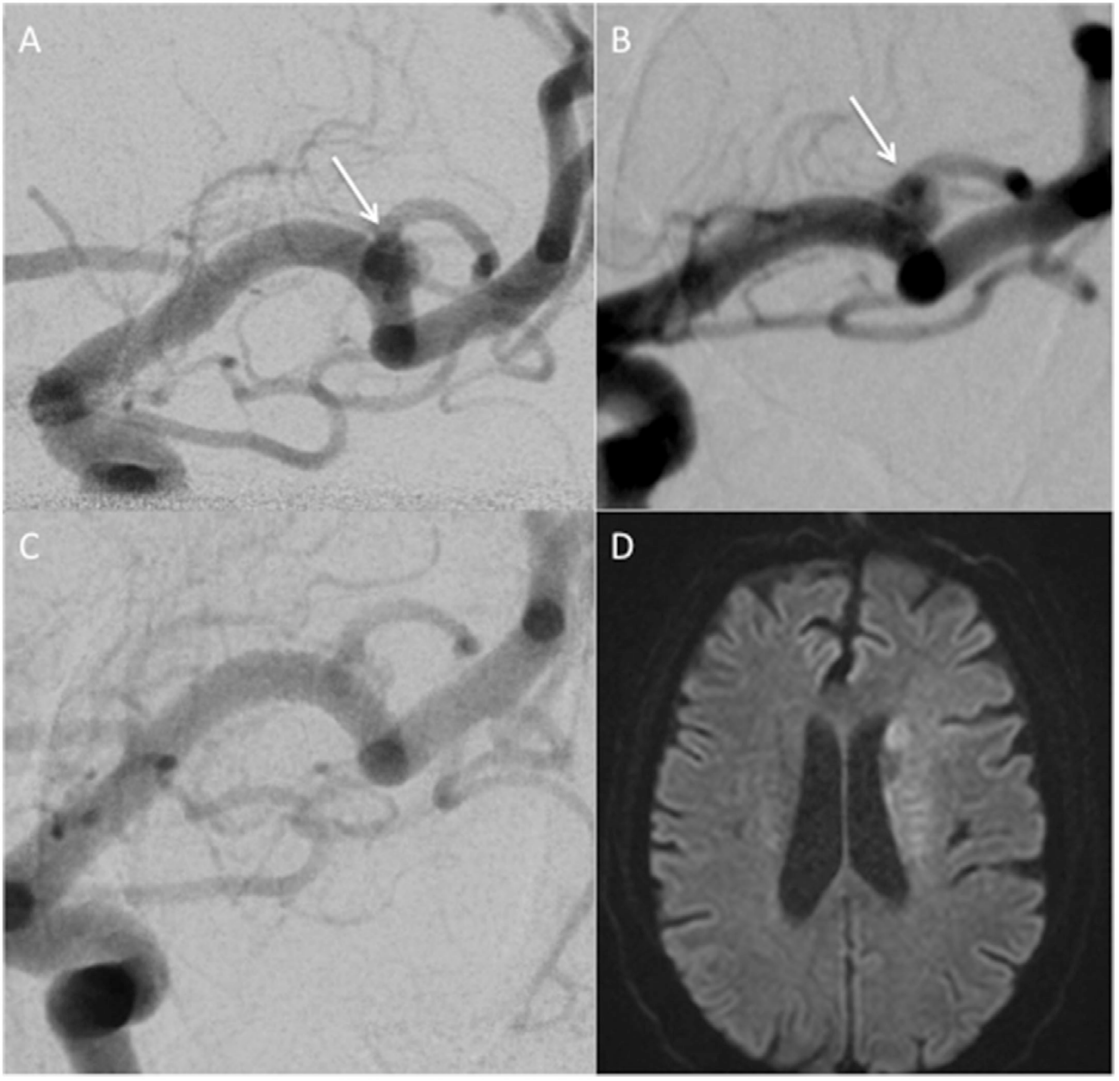
**A patient treated with a single p64 FDS for a middle cerebral artery bifurcation anerurysm**. An incidental untreated bifurcation aneurysm **(A)**. The same aneurysm in a different projection shows a wide necked saccular MCA bifurcation **(B)**. There were no intra-operative complications and immediately post-procedure the patient awoke with baseline neurology. Five days post-procedure, the patient developed a right hemiparesis. Catheter angiography at this time **(C)** showed all branches were patent with anterograde flow and minimal filling of the aneurysm. An MRI showed restricted diffusion **(D)**.

There was one case of an immediate thrombosis of the FDS, which resolved secondary to intra-arterial infusion of Eptifibatide and the patient recovered without neurological sequelae. One patient developed hemiparesis four days after the implantation of the FDS. The emergent MRI showed restricted diffusion in the basal ganglia and centrum semiovale. The immediate angiogram showed no in-stent thrombosis or vessel occlusion. Although the exact cause of the infarction in this case is not know, it is possible that a drop in blood pressure plus reduced flow through the FDS may have resulted in decreased perfusion to the infarcted region (Figure 2).

## Discussion

The MCA is the larger of the two terminal branches of the internal carotid artery. Over its course the MCA is divided into four segments: the M1 (sphenoidal) segment, the M2 (insular) segment, the M3 (opercular) segment, and the M4 (cortical) segments (21). The branching pattern of the main trunk of the MCA is variable with a bifurcation being the most common: absence of a main division in 6%, bifurcation in 64%, trifurcation in 29%, and quadrifurcation in 1% of people. The location of the bifurcation depends on the length of the M1 Segment. The main divisions are of equal diameter in only 15% of the cases with the inferior trunk being dominant in 50% (22).

Aneurysms of the MCA occur most often at the main division point (23). In a review of a population-based series of 3,005 patients with 4,253 intracranial aneurysms, 1,704 were MCA aneurysms, of which 1,385 (81%) were located at the main bifurcation of the MCA. The percentage of aneurysms located at the main bifurcation was even higher among ruptured MCA aneurysms (89%) (2). Elsharkawy et al. (24) analyzed 1,009 aneurysms located on the MCA to determine if there were particular risk factors associated with a risk of rupture. In this study, location at the MCA bifurcation, wall irregularity, and non-spherical shape were all identified as risks for rupture. Interestingly, it was also seen that 26% of patients with ruptured MCA bifurcation aneurysms were classified as small (<7 mm) and that this was significantly greater than the percentage of patients with large (15–24 mm) and giant (≥25 mm) aneurysms, which presented with rupture in 15 and 2% of cases, respectively. This highlights, that at least in this location, small aneurysms carry a not insignificant risk of rupture.

From an anatomical perspective MCA bifurcation aneurysms are often wide necked and may incorporate one or more branches. This can be challenging for traditional endovascular approaches such as coiling without adjunctive devices (2, 25, 26). In the study of Jin et al. (27), 103 aneurysms of the MCA bifurcation were treated with only coiling. The post-coiling angiogram in these patients showed a complete occlusion in 27.2% and residual neck in 58.3%. At follow-up angiography (mean 30 months), recanalization was seen in 25% of cases and just over 20% of these recanalized aneurysms were deemed completely occluded on post-coiling angiography. Other authors have also published their experience of MCA aneurysm treatment involving endovascular techniques; for example, Bracard et al. reviewed 152 MCA aneurysms treated by coil occlusion. They showed complete or near-complete occlusion in 84.2% of aneurysms, of which 80.2% persisted at the 5-year follow-up (28). In a large retrospective analysis of 300 consecutive endovascularly treated MCA aneurysms (80% ruptured), complete or near-total occlusion was achieved in 91% of cases (25). Similarly, Zaidat et al. (29) reviewed their endovascular treatment of 161 consecutive MCA aneurysms and reported technical feasibility of 98.8% and a retreatment rate of 11%. In the meta-analysis of 12 studies performed by Brinjikji et al., they noted an overall occlusion rate (complete or near-complete occlusion) of 82.4% with an overall procedure-related permanent morbidity/mortality rate of 5.1% for unruptured aneurysms. While these latter studies are useful, a major difficulty in their interpretation is they include all MCA aneurysms together with no separation based on anatomical location.

In order to address the various anatomical abnormalities that may be encountered when treating MCA bifurcation aneurysms, various techniques have been developed and include: balloon remodeling (3–7), stent-assisted coiling (7–9, 11, 12), neck-bridging devices (30, 31), and intra-aneurysmal flow disrupters (32–35).

Eboli et al. (36) recently published their series of 184 MCA bifurcation aneurysms treated endovascularly. In this series, stent-assisted coiling was required in 70 cases (38%), and only 3 cases (1.6%) required “Y” stenting. They achieved an initial total occlusion rate of 59.8%; however, on follow-up angiography (mean 41 months) complete aneurysm occlusion was seen in 90.1% of patients with a further 6.1% of patients having a stable remnant. The overall peri-procedural morbidity was 3.8% and the mortality rate in the unruptured cohort was 0%.

The Woven EndoBridge [WEB (Sequent Medical, Aliso Viejo, CA, USA)] is an intra-saccular device designed to disrupt the intra-aneurysmal flow at the level of the neck to encourage intra-aneurysmal thrombosis. Although some authors reported high feasibility of this device with promising early and midterm post-procedural angiographic results (32–34), the follow-up study by Cognard and Januel reported worsening at follow-up in 71.5% in their series of 14 patients with wide-neck bifurcation aneurysms (of which 11 were at the MCA bifurcation) with compression of the cage demonstrated in 8 of the 14 cases (37). Additionally, the available sizes of the WEB device may render it unsuitable to small wide-necked aneurysms. The pCONus (Phenox, Bochum, Germany) device is an electrolytically detachable self-expanding nitinol implant with four distal petals designed to open inside the aneurysm providing support for coiling of wide-neck bifurcation aneurysms. Aguilar-Pérez et al. recently published their experience with the pCONus neck-bridging device for the treatment of wide-necked aneurysms. This series included 11 MCA aneurysms with complete occlusion of 6 aneurysms (54.5%) at follow-up and only neck remnant in 3 cases (27.2%) (30).

Since their introduction, flow diverter stents (FDS) have gained popularity for the treatment of intracranial aneurysms. The occlusion of an aneurysm by FDS is initially by the promotion of intra-aneurysmal thrombosis through induced flow stasis. The blood’s flow though the struts of a FDS depends on flow demands and thus preserving the patency of covered branches where flow is needed (38). For example, even relatively small arteries such as the anterior choroidal artery remain patent after coverage by FDS as was recently shown by Neki et al. (39). Covered branches with an adequate collateral arterial supply, such as the ophthalmic artery, where the orbit enjoys a rich collateral blood supply through branches of the external carotid artery, frequently undergo spontaneous, asymptomatic occlusion (40). Rangel-Castilla et al. recently published their longer-term results of 82 aneurysms treated with FDS with an emphasis on covered branches (41). In this study of 76 covered ophthalmic arteries, 10.5% were occluded (clinically asymptomatic), 28 posterior communicating arteries were covered with 10.7% of the vessels occluded (clinically asymptomatic), and 21 covered anterior choroidal arteries with no evidence of occlusion. This study also reported two cases where the anterior cerebral artery was covered and there was occlusion with filling of the A2 segment *via* the contralateral anterior cerebral artery. Therefore, there is evidence that branch occlusion can be asymptomatic. However, this may not necessarily be the case in more distal locations such as at the MCA bifurcation.

Yavuz et al. (42) used the PED to treat 25 MCA aneurysms located at or distal to the bifurcation in 21 patients and reported a complete occlusion in 21 of the cases (84%). There were no mortalities, and apart from one patient who developed ischemia several days after the procedure (which was attributed to vasospasm by the authors), there were no significant peri-procedural morbidities. There was reduced filling of six and total occlusion of three of the covered branches, all of which remained clinically asymptomatic. The authors concluded that the PED is a safe and feasible option in treating aneurysms arising at the MCA bifurcation. The results reported by Caroff et al. (43) were not so promising. In their retrospective review of 14 patients harboring 15 saccular MCA bifurcation aneurysms treated with FDS, the authors reported ischemic complications in 43% of the cases detected on MRI, and although, there were no mortalities, procedure-related morbidities reached 21% on follow-up, most of these were related to occlusion or slow flow in the covered branches. Complete occlusion was, however, achieved in only 62% of the treated aneurysms. In another report by Briganti et al. (44), total occlusion of the MCA bifurcation aneurysms was achieved in 80% with the rest of the aneurysms being partially occluded. This series included 14 patients harboring 15 MCA aneurysms of which 13 were located at the MCA bifurcation, all of which were treated using the PED. Of the 13 side branches covered by the PED, follow-up angiographic studies showed reduced flow in 6 and total occlusion of 3 of them. The authors reported ischemic complications in 27% of the cases with permanent neurological deficient in 21%. Topcuoglu et al. (45) recently published their series of 29 aneurysms of the MCA treated with flow diversion, 6 of which occurred at the true bifurcation of the MCA (defined as the division of the MCA in superior and inferior trunks). Of these 6 cases, all of which were treated with the Silk FDS (Balt, Montmercy, France), occlusion of the covered branch occurred in 50% of cases, none of which resulted in morbidity or mortality. In our series, we achieved a complete occlusion in 50% of patients and near-complete occlusion in 41.7%. We had no mortalities and only one case of permanent morbidity. We believe, as with flow diversion used in other sites, that the aneurysms with near-complete occlusion will continue to occlude over time as neo-endothelialization occurs; however, longer-term follow-up is required. In a single case, the aneurysm showed no change at delayed angiography (9 months), and we are unable to explain this phenomenon. The patient had no background medical conditions that could potentially interfere with neo-endothelialization nor did they demonstrate any abnormal response to the antiplatelet medication.

Saleme et al. (46) sought to clarify the role of covered branches and collateral supply. They compared remodeling of the side braches covered after the deployment of flow diverters dividing the aneurysms in two groups based on whether the territory supplied by the side branch received a direct collateral supply or not. This study included the placement of FDS across the anterior cerebral artery, the anterior communicating artery, the terminal ICA, and the MCA (51.4% of cases). They showed that in the group with a direct collateral supply 78.5% of covered branches had undergone narrowing or occlusion at 6 months, although no new strokes were seen on MR imaging. Hence, the authors suggest that symptomatic remodeling of covered side braches depends on the extent and type of collateral supply, and this is similar to the findings reported earlier by Rangel-Castilla et al. (39–41). One explanation of the lower occlusion rate observed with flow diversion for bifurcation aneurysms might be the persistent flow through the covered braches incorporated by the aneurysm. Fahed et al. (47) compared flow diversion with and without occlusion of the jailed branch in 14 wide-neck aneurysms induced in 8 canines and found that occlusion of the jailed branch resulted in better occlusion rates of aneurysmal occlusion. Patent aneurysms were associated with leaks or holes in the neo-intima covering the aneurysm neck. The authors concluded that persistent flow to the jailed branch is a potential cause of treatment failure after flow diversion for bifurcation aneurysms. Furthermore, it is also worth remembering that the porosity of FDS is known to alter with curves and this appears to be most marked for FDS deployed across bifurcation aneurysms and on the outer edge curves of vessels (48, 49). This effect has been studied for both the PED and the Silk flow diverters with similar results and it is likely that the same effect will be seen with the p64.

Despite the plethora of currently available modality for the treatment of MCA aneurysms, considering the relatively straight forward access, the good outcome after clipping and the ability for simultaneous removal of a space occupying hematoma in cases of ruptured aneurysms, a surgical approach remains the principal treatment of MCA aneurysms in many centers (2, 50, 51). In their series of 282 ruptured and 261 unruptured MCA aneurysms, Rodríguez-Hernández and colleagues reported complete aneurysm obliteration in 98.3% and good clinical outcomes in 92% of patients with unruptured and 70.2% with ruptured aneurysms (52). In a review of the literature by Yang and Haung, the authors favored the microsurgical approach for the definitive management of MCA aneurysms. The reviewed surgical series reported occlusion rates between 90 and 89.3% with good clinical outcomes in 92–100% of unruptured and 70–80% of ruptured MCA aneurysms treated with clipping. The authors also suggested that the significantly higher retreatment rated and lower occlusion rates of the endovascular approach would offset the favorable clinical outcome in the short term of the less invasive approach (53).

Our study has several limitations. The sample size is small and the treated aneurysms are heterogeneous. Another limiting factor is that flow diversion was not the primary treatment in six cases (42.8%), rather implemented as a secondary therapy for persistent aneurysmal perfusion after coiling or clipping. The results are also limited by the relatively short follow-up period (13 months on average), especially because the definitive results of this modality depend on the progressive reduction of flow inside the aneurysmal sac. Although one type of flow diverter stents, namely the p64, was used in all but one case in our series, we believe that the relatively inferior results in comparison to other endovascular or surgical modalities are related to the special anatomic consideration of the MCA bifurcation, rather than the technical specification of the flow modulation device.

## Conclusion

Based on our small series, flow diversion for the treatment of bifurcation MCA aneurysms is feasible with good angiographic results and acceptable complication rates. However, compared to the results of other endovascular techniques and to surgery, total occlusion of bifurcation MCA aneurysms seems to be less frequent with flow diversion. Therefore, we believe that flow diversion should be reserved for cases where other treatment modalities are deemed unfeasible or carry excessive risk.

## Ethics Statement

As this study was retrospective in design and all identifying information removed from images and data, local ethics committee approval was not required. All patients gave informed consent for the procedure. Consent for publication was not required and all identifiable information removed from the manuscript.

## Author Contributions

PB—data collection, imaging review, manuscript preparation; MA—data collection, imaging review, manuscript preparation; HB—manuscript editing, language correction; HH—manuscript editing, manuscript preparation, concept; OG—manuscript editing, concept; and MAP—guarantor.

## Conflict of Interest Statement

MAP and PB serve as proctors and consultants for phenox GmbH with moderate financial compensation. HH is a co-founder and shareholder of phenox GmbH. The other authors declare no conflict of interest.
